# Diagnostic Value of Magnetic Resonance Spectroscopy in Radiation Encephalopathy Induced by Radiotherapy for Patients with Nasopharyngeal Carcinoma: A Meta-Analysis

**DOI:** 10.1155/2016/5126074

**Published:** 2016-02-03

**Authors:** Wang-Sheng Chen, Jian-Jun Li, Lan Hong, Zeng-Bao Xing, Fen Wang, Chang-Qing Li

**Affiliations:** ^1^Department of Radiology, People's Hospital of Hainan Province, Haikou 570311, China; ^2^Department of Gynaecology, People's Hospital of Hainan Province, Haikou 570311, China; ^3^Department of Radiotherapy, People's Hospital of Hainan Province, Haikou 570311, China

## Abstract

In this study, articles in English and Chinese were selected from available electronic databases prior to September 2014. The metabolic concentrations and patterns of N-acetylaspartic acid (NAA), Choline (Cho), Creatine (Cr), NAA/Cr, NAA/Cho, and Cho/Cr ratios in radiotherapy-induced radiation encephalopathy by proton magnetic resonance spectroscopy were extracted. A meta-analysis was performed to quantitatively synthesize findings of these studies. Weighted mean difference (WMD) and 95% confidence intervals (95%CIs) were calculated using random or fixed effective models. Heterogeneity between studies was assessed using the Cochrane *Q* test and *I* ^2^ statistics. The results indicated that a total of 4 researches involving 214 patients met inclusion criteria. Depending on methodologies of selected studies, control groups were referred to as healthy subjects. The combined analysis revealed that there was no significant difference in value of Cr between radiotherapy group and healthy control group (WMD = −1.483, 95% CI: −67.185–64.219, *p* = 0.965). However, there were significant difference in values of NAA (WMD = −18.227, 95%CI: −36.317–−0.137, *p* = 0.048), Cho (WMD = 38.003, 95%CI: 5.155–70.851, *p* = 0.023), NAA/Cr (WMD = −1.175, 95%CI: −1.563–−0.787, *p* = 0.000), NAA/Cho (WMD = −1.108, 95%CI: −2.003–0.213, *p* = 0.015), and Cho/Cr (WMD = −0.773, 95%CI: 0.239–1.307, *p* = 0.005). In conclusion, MRS can be regarded as an effective and feasible imaging test for radiotherapy-induced radiation encephalopathy in NPC patients.

## 1. Introduction

Nasopharyngeal carcinoma (NPC) is a common cancer in East Asia with a regional difference character [1]. The incidence of this disease is 20 to 30 per 100,000 persons in southeast Asia, while there is less than 1 per 100,000 in western countries. The incidence, especially, ranges from 15 to 50 per 100,000 in southern China [2]. Therefore, it is of great importance to study this disease.

There are various treatment strategies for NPC, including surgery, chemotherapy, radiotherapy, and target therapy. Due to the anatomical character and high sensitivity to radiation, the radiotherapy serves as the preferential strategy for nasopharyngeal carcinoma patients. However, the application of radiotherapy in the NPC treatment always comes with several severe complications. Among the complications, the radiation encephalopathy (REP) is one of the common and serious radiotherapy-related complications [3, 4]. The effective way to treat REP is to detect this disease in early stage. Since there are no specific symptoms during the early period of REP, imaging examinations, including CT and MRI, become the major methods to monitor the incidence of REP. However, the diagnostic capabilities of CT or MRI were not satisfactory; the disease would become irreversible when there were visible changes by these imaging examinations. Therefore, the detection of REP in the early stage would help reduce the incidence of this serious complication and contribute largely to better prognosis for the NPC patients [5].

Magnetic resonance spectroscopy (MRS), a noninvasive imaging technique, could monitor the biologic characteristics and metabolism status of lively tissues [6–8]. And this technique has shown promise in the evaluation of disease at an early stage [9, 10]. Our preliminary experience with MRS has been obtained for diagnosis of radiation injury of normal brain tissue following radiotherapy, in which the NAA, Cr, and Cho and the ratios of NAA/Cr, NAA/Cho, and Cho/Cr can vary greatly. Therefore, we choose the above molecules to evaluate the diagnostic value of MRS in patients. Some studies had investigated the significance of MRS in the detection of radiotherapy-induced REP [11–13]. However, a meta-analysis of published data in this field is also seldom. Given the importance of an early detection of REP in the NPC patients and that there are no concurrent studies concerning the application of MRS in the NPC patients developing REP, we performed a meta-analysis to determine the accuracy and significance of MRS in the evaluation of the REP in the NPC patients who underwent the radiotherapy.

## 2. Materials and Methods

### 2.1. Data Source and Study Selection

We systematically searched the electronic databases both in English and Chinese prior to September 1, 2014, including The Cochrane Library, PUBMED, China National Knowledge Infrastructure and Wanfang Database. There was no limit for the starting data. The searching strategy was conducting the searching by combining the terms “magnetic resonance spectroscopy (MRS),” “nasopharyngeal carcinoma,” “radiation encephalopathy”/“radiation brain injury,” and “radiotherapy” using [Mesh] or [Keyword]. Potentially relevant articles were screened by two independent reviewers. Disagreements were resolved by discussion or upon consensus from a third reviewer.

### 2.2. Inclusion and Exclusion Criteria

The inclusion criteria were as follows: (1) pathological diagnosis of nasopharyngeal carcinoma; (2) patients who underwent radiotherapy; (3) published studies comparing the application of MRS in the NPC patients and healthy controls; (4) available NAA, Cr, Cho, NAA/Cr, NAA/Cho, and Cho/Cr data for each group. Studies, such as review articles, case reports, or animal studies, were excluded. And studies without controls and complete data were also excluded. Two reviewers independently judged the eligibility of the studies. Disagreements between reviewers were resolved by consensus from the third reviewer.

### 2.3. Data Extraction

The data extraction and quality assessment were performed independently by two reviewers. We extracted data including the authors, publication year, the number of patients, the number of controls, and the values of the parameters including NAA, Cr, Cho, NAA/Cr, NAA/Cho, and Cho/Cr data.

### 2.4. Statistical Analysis

The data were analyzed using Stata software (Version 12.0). Weight mean differences (WMDs) and 95% confidence intervals (95% CIs) were used to analyze continuous data and were analyzed by the Stata software. For the WMDs calculation, the standard deviation individual was used. The *Q* statistic of the Chi-square value test and the inconsistency index (*I*-squared, *I* ^2^) were used to measure the heterogeneity of the included individual studies. And *p* < 0.1 or *I* ^2^ > 50% suggested that there was notable heterogeneities, which suggested that the test performance was summarized by using a random-effects coefficient binary regression model; otherwise, a fixed-effects coefficient binary regression model was used. The presence of publication bias was visually assessed by performing a Deeks funnel plot and an asymmetry test with the Stata software. Publication bias was considered to be present if there was a nonzero slope coefficient (*p* < 0.05).

## 3. Results

### 3.1. The Characteristics of Included Studies

According to the searching terms “nasopharyngeal carcinoma,” “radiotherapy,” “magnetic resonance spectroscopy,” and “radiation brain injury”/“radiation encephalopathy,” a total of 10 papers in English and 189 papers in Chinese were screened from the databases. After screening the abstracts and removing the studies without healthy individuals as controls, only 4 studies with complete available data were included in this study. The included studies involved 148 participants with nasopharyngeal carcinoma and 66 healthy controls. And the characteristics of the included studies were summarized in Table 1.

### 3.2. Data Analysis

**Figure 1:**
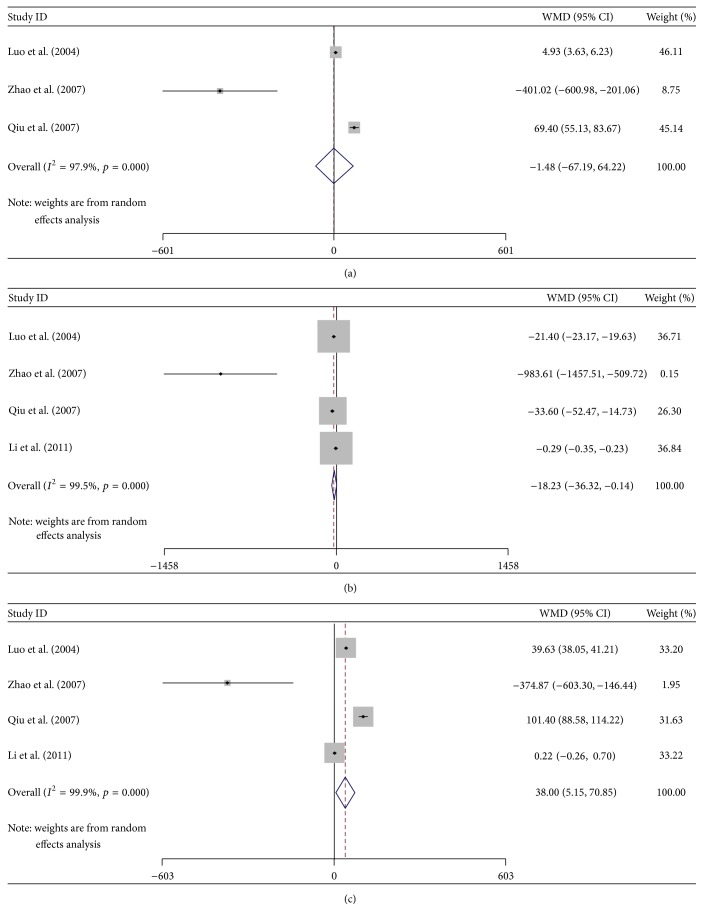
The changes of MRS related metabolic parameters compared between the radiotherapy group and control group analysed by Forest Plot. (a) The value of Cr; (b) the value of NAA; (c) the value of Cho.

**Figure 2:**
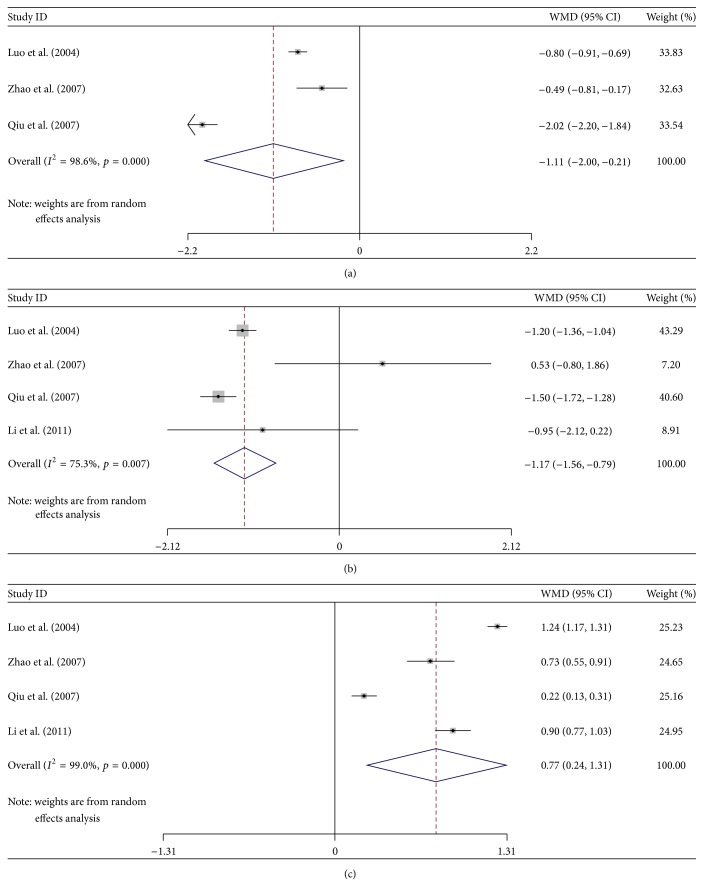
Forest Plot analysis showed the changes of MRS related metabolic parameter ratios compared between the radiotherapy group and control group. (a) The value of NAA/Cho; (b) the value of NAA/Cr; (c) the value of Cho/Cr.

We extracted the important parameters of MRS detection from the included studies, including the values of N-acetylaspartic acid (NAA), Choline (Cho), Creatine (Cr), NAA/Cr, NAA/Cho, and Cho/Cr. We analyzed the significances of these parameters in the NPC patients who underwent radiotherapy. There were significant heterogeneities between the included studies for all the examined parameters (*I* ^2^ > 75%, *p* < 0.01, data not shown); accordingly, a random effective model was used for the meta-analysis. The random effective model analysis showed that there was no significance between the NPC patients and healthy controls in the concentration of Cr (WMD = −1.483, 95% CI: −67.185–64.219, *p* = 0.965, Figure 1(a)), which indicates that Cr might not work well as an important parameter in detecting the early stage of REP. However, the meta-analysis indicated that the values of NAA (WMD = −18.227, 95% CI: −36.317–−0.137, *p* = 0.048), Cho (WMD = 3 8.003, 95% CI: 5.155–70.851, *p* = 0.023), NAA/Cr (WMD = −1.175, 95% CI: −1.563–−0.787, *p* = 0.000), NAA/Cho (WMD = −1.108, 95% CI: −2.003–0.213, *p* = 0.015), and Cho/Cr (WMD = −0.773, 95% CI: 0.239–1.307, *p* = 0.005) exerted more advantage in diagnosing the REP at an early stage (Table 2). The value of NAA was slightly decreased, while the value of Cho was slightly increased in the radiotherapy group. The NAA/Cho and NAA/Cr ratios decreased significantly after radiotherapy compared with the healthy controls, while the Cho/Cr ratio increased significantly compared with the healthy control (Figure 2). These results indicated that the MRS could be used as an effective way in diagnosing REP at an early stage by monitoring the metabolic parameters.

A meta-regression analysis was used to determine whether the variables were associated with the effect, as no heterogeneity has to be explained (*I* ^2^ = 0 percent). From the meta-analysis analysis, we found that the heterogeneity mainly occurs in the “NAA” of Zhao et al.'s study (with higher *I* ^2^ = 78%) and “Cho” of Zhao et al.'s study (with higher *I* ^2^ = 91.5%) (Supplementary Table 1 in Supplementary Material available online at http://dx.doi.org/10.1155/2016/5126074). Therefore, the heterogeneity may be caused by [15].

## 4. Discussion

Currently, radiotherapy is the standard therapy for nasopharyngeal carcinoma patients [18]. And there is a positive relationship between the radiation dose and the radiation effect. Gaining a better prognosis means that there was much chance to develop severe complications. Among the radiotherapy-related complications, radiation-induced brain injury is a common and serious disease [3, 4], which occurs in 5~24% of NPC patients with radiotherapy [5]. Currently, the mechanisms of radiation-induced brain injury were not generally concluded. Generally, the underlying mechanisms may include the nerve and glial cell injury and the demyelination, softening, and the necrosis of white matter. The radiation-induced REP may be repaired at a certain early stage and to a certain extent. Therefore, it is of great importance to detect the REP at an early stage.

Conventional imaging tests, such as computed tomography and MRI, can only demonstrate severe and irreversible brain damage during the late delayed reaction phase, whereas MRS can monitor brain damage at the molecular level by assessing the metabolic concentration trends during the early delayed reaction period [6, 7, 19]. MRS is a noninvasive diagnostic tool, which could detect the biochemical and metabolic changes in the live tissues [5, 9, 19]. MRS evaluates the functions of tissues by measuring the concentrations of NAA, Cr, Cho, NAA/Cr, NAA/Cho, and Cho/Cr [7, 19]. There are several studies that tracked the incidence of REP by measuring the values of these parameters and compared them with the normal healthy controls [14–17]. Li et al. found that the value of Cho, Cho/Cr in the temporal lobe injury was much higher in the radiotherapy group than the normal healthy group, while the values of NAA, NAA/Cr, and NAA/Cho were much lower in the radiotherapy group than the normal healthy controls [17]. Luo et al. demonstrated that the peak value and rations of NAA, Cr, Cho, NAA/Cr, NAA/Cho, and Cho/Cr were different among different areas of the brain, and there were changes in these parameters between the radiotherapy group and healthy control group [14]. Zhao et al. [15] also studied the characters of MRS-related parameters in detecting the radiation induced temporal lobe injury for the NPC patients. Significantly decreased NAA, Cho, Cr, and NAA/Cho ratios were detected in the radiotherapy group, while significant elevated Cho/Cr ration was measured. Qiu et al. [16] found that the peak value of NAA decreased slightly, while the Cr and Cho values decreased significantly or even became absent in the patient group. Meanwhile, the NAA/Cr and NAA/Cho ratios in the radiotherapy group were decreased significantly compared with the control group [16, 20]. Based on these studies, we performed meta-analysis to assess the utility of MRS in the diagnosis of radiation-induced brain damage for the nasopharyngeal carcinoma patients. We extracted the metabolite parameters of MRS, which include NAA, Cho, Cr, NAA/Cr, NAA/Cho, and Cho/Cr ratios. There were significant heterogeneities among all the included studies. These heterogeneities might be due to the lack of large-scaled random control studies and the small size of included population. Based on the heterogeneity test, a random effective analysis was performed. Unlike the other studies, there was no significant difference in the concentration of Cr between the radiotherapy group and control groups, whereas here there were slight differences in the value of NAA and Cho. These results demonstrated that there were limitations when detecting the REP only based on the values of single parameter. We further assessed the utilities of the ratios, and it turned out that there were significantly reduced ratios of NAA/Cho and NAA/Cr in the radiotherapy group, while there was increased ratio of Cho/Cr in the radiotherapy group. These results indicated that the metabolic parameter ratios NAA/Cho, NAA/Cr, and Cho/Cr might be more effective in monitoring the incidence of radiation-induced brain injury.

However, to some extent, there are limitations of this meta-analysis which needed to be addressed. Firstly, there was no available detailed individual data and a more precise subgroup analysis should be performed on other variables such as age, sex, and stage of the disease. Secondly, the sample sizes of the 4 included studies were rather small and not adequate enough to confirmedly assess the utilities of MRS in the detection of radiation-induced brain injury at an early stage. Thirdly, we included only published studies in this study; the unpublished data or clinical trials have not been included in this analysis. Fourthly, due to the number limitation of the included studies, there was existence of publication bias in some comparisons, which could potentially influence the results of our meta-analysis. Fifthly, there was high heterogeneity in our study. Though we have found and explained the heterogeneity [15], which is the problem arising from the cited study. In the following study, we would adjust the heterogeneity to avoid the heterogeneity.

In summary, this meta-analysis suggests that the MRS could be an effective way in detecting the radiation encephalopathy by monitoring the changes of the metabolic parameters. More future large-scaled studies are still needed to confirm these results.

## Supplementary Material

From the meta-analysis analysis, we found that the heterogeneity mainly occurs in the “NAA” of Zhao et al.'s study (with higher *I* ^2^ = 78%) and “Cho” of Zhao et al.'s study (with higher *I* ^2^ = 91.5%) (Supplementary Table 1). Therefore, the heterogeneity may be caused by the reference of Zhao et al. (2007).

## Figures and Tables

**Table 1:** Characteristics of studies included in the meta-analysis.

Study	Year	Language	Control source	Number of patients in radiotherapy group	Number of healthy controls in control group
Luo et al. [14]	2004	English	Healthy controls	76	25
Zhao et al. [15]	2007	Chinese	Healthy controls	23	21
Qiu et al. [16]	2007	English	Healthy controls	21	10
Li et al. [17]	2011	Chinese	Healthy controls	28	10

**Table 2:** Results from meta-analysis compared the metabolic parameters between the radiotherapy group and control group.

NAA		Cr		Cho		NAA/Cr		NAA/Cho		Cho/Cr	
WMD (95% CI)	*p*	WMD (95% CI)	*p*	WMD (95% CI)	*p*	WMD (95% CI)	*p*	WMD (95% CI)	*p*	WMD (95% CI)	*p*
−18.227 (−36.317, −0.137)	0.048	−1.483 (−67.185, 64.219)	0.965	38.003 (5.155, 70.851)	0.023	−1.175 (−1.563, −0.787)	0.000	−1.108 (−2.003, −0.213)	0.015	0.773 (0.239, 1.307)	0.005
