# Supplementary material for: Diagnostic Value of Magnetic Resonance Spectroscopy in Radiation Encephalopathy Induced by Radiotherapy for Patients with Nasopharyngeal Carcinoma: A Meta-Analysis
Source: Biomed Res Int. 2016 Feb 3;2016:5126074. doi: 10.1155/2016/5126074 (PMC4756187; doi:10.1155/2016/5126074)
Supplement: Supplementary file 1 — From the meta-analysis analysis, we found that the heterogeneity mainly occurs in the “NAA” of Zhao et al.'s study (with higher I 2 = 78%) and “Cho” of Zhao et al.'s study (with higher I 2 = 91.5%) (Supplementary Table 1). Therefore, the heterogeneity may be caused by the reference of Zhao et al. (2007). [file 5126074.f1.doc]

|  | Relative Risk (95% CI; I2) | Explained Heterogeneity (P value) |
| --- | --- | --- |
| NAA |  |  |
| Luo et al,2004 | -21.4 (-23.17, -19.63) | 7.84% (0.912) |
| Zhao et al, 2007 | -983.61 (-1457.51, -509.72) |  |
| Qiu et al, 2007 | -33.6 (-52.47, -14.73) |  |
| Li et al, 2011 | -0.29 (-0.35, -0.23) |  |
| Cr |  |  |
| Luo et al,2004 | 4.93 (3.63, 6.23) | No heterogeneity (0.747) |
| Zhao et al, 2007 | -401.02 (-600.98, -201.06) |  |
| Qiu et al, 2007 | 69.4 (55.13, 83.67) |  |
| Li et al, 2011 | -1.48 (-67.19, 64.22) |  |
| Cho |  |  |
| Luo et al,2004 | 39.63 (38.05, 41.21) | 5.62% (0.675) |
| Zhao et al, 2007 | 1374.87 (-603.3, -146.44) |  |
| Qiu et al, 2007 | 101.4 (88.58, 114.22) |  |
| Li et al, 2011 | 0.22 (-0.26, 0.7) |  |
| NAA/Cr |  |  |
| Luo et al,2004 | -1.2 (-1.36, -1.04) | No heterogeneity (0.817) |
| Zhao et al, 2007 | 0.53 (-0.8, 1.86) |  |
| Qiu et al, 2007 | -1.5 (-1.72, -1.28) |  |
| Li et al, 2011 | -0.95 (-2.12, 0.22) |  |
| NAA/Cho |  |  |
| Luo et al,2004 | -0.8 (-0.91, -0.69) | No heterogeneity (0.645) |
| Zhao et al, 2007 | -0.49 (-0.81, -0.17) |  |
| Qiu et al, 2007 | -2.02 (-2.2, -1.84) |  |
| Li et al, 2011 | -1.11 (-2, -0.21) |  |
| Cho/Cr |  |  |
| Luo et al,2004 | 1.24 (1.17, 1.31) | No heterogeneity (0.905) |
| Zhao et al, 2007 | 0.73 (0.55, 0.91) |  |
| Qiu et al, 2007 | 0.22 (0.13, 0.31) |  |
| Li et al, 2011 | 0.9 (0.77, 1.03) |  |

**Table 1.** Results Meta-Analysis and Meta-Regression.
